# Statistics of the Bifurcation in Quantum Measurement

**DOI:** 10.3390/e21090834

**Published:** 2019-08-26

**Authors:** Karl-Erik Eriksson, Kristian Lindgren

**Affiliations:** Complex Systems Group, Department of Space, Earth and Environment, Chalmers University of Technology, SE-412 96 Gothenburg, Sweden

**Keywords:** quantum measurement, scattering theory, statistics, Born’s rule

## Abstract

We model quantum measurement of a two-level system μ. Previous obstacles for understanding the measurement process are removed by basing the analysis of the interaction between μ and the measurement device on quantum field theory. This formulation shows how inverse processes take part in the interaction and introduce a non-linearity, necessary for the bifurcation of quantum measurement. A statistical analysis of the ensemble of initial states of the measurement device shows how microscopic details can influence the transition to a final state. We find that initial states that are efficient in leading to a transition to a final state result in either of the expected eigenstates for μ, with ensemble averages that are identical to the probabilities of the Born rule. Thus, the proposed scheme serves as a candidate mechanism for the quantum measurement process.

## 1. Introduction

Quantum mechanics is at the basis of all modern physics and fundamental for the understanding of the world that we live in. As a general theory, quantum mechanics should apply also to the measurement process. From the general experience of non-destructive measurements, we draw conclusions about the interaction between the observed system and the measurement apparatus and how this can be described within quantum mechanics.

We thus consider a quantum system μ, interacting with a measurement device. For simplicity we assume that μ is a two-level system that is not destroyed in the process. Then after the measurement, μ ends up in one of the eigenstates of the measured observable. If μ is prepared in one of these eigenstates, it remains in that state after the measurement. If μ is initially in a superposition of the two eigenstates, it still ends up in one of the eigenstates and the measurement result is the corresponding eigenvalue. The probability for a certain outcome is the squared modulus of the corresponding state component in the superposition (Born’s rule).

An essential question is whether the probabilistic nature of quantum measurement with Born’s rule is an inherent feature of quantum mechanics or whether it can be shown to hold as a result of a quantum-mechanical treatment of the measurement process combined with a statistical analysis. In the latter case, a single measurement would be a quantum-mechanical process in which the state of the measurement apparatus (possibly including its surroundings) determines the result. Born’s rule would then emerge from the statistics of the ensemble that describes the measurement apparatus in interaction with the system subject to measurement.

The requirement that μ, if initially in an eigenstate of the observable, remains in that eigenstate after interacting with the apparatus, is usually considered to lead to a well-known dilemma: If applying the (linear) quantum mechanics of the 1930s to μ in an initial superposition of those eigenstates, the result of the process appears to be a superposition of the two possible resulting states for μ and the apparatus without any change in the proportions between the channels. This has been referred to as von Neumann’s dilemma [1], and it has led to paradoxical conclusions such as Schrödinger’s cat.

Attempts to get around this problem include Everett’s relative-state formulation [2] and its continuation in DeWitt’s many-worlds interpretation [3] as well as non-linear modifications of quantum mechanics [4,5,6,7,8]. In the non-linear modifications, one gets the bifurcation of the measurement process but the non-linear character of the basic theory introduces new conceptual difficulties. Mathematically our treatment can be seen to be very close to quantum diffusion [5]; we have chosen to follow the same conventions in handling the statistics of stochastic variables as in Ref. [5]. The ambition to understand quantum measurement as a deterministic process we share with the De Broglie-Bohm theory [9], with the difference that we look for how details in the measurement device influence the process.

Bell pointed out that the Everett-DeWitt theory does not properly reflect the fact that the presence of inverse processes and interference are inherent features of quantum mechanics [10]:

Thus, DeWitt seems to share our idea that the fundamental concepts of the theory should be meaningful on a microscopic level and not only on some ill-defined macroscopic level. However, at the microscopic level there is no such asymmetry in time as would be indicated by the existence of branching and the non-existence of debranching. [...] [I]t even seems reasonable to regard the coalescence of previously different branches, and the resulting interference phenomena, as the characteristic feature of quantum mechanics. In this respect an accurate picture, which does not have any tree-like character, is the ’sum over all possible paths’ of Feynman.

Therefore, as suggested by Bell, we investigate work of Feynman for a correct theory. We choose the scattering theory of quantum field theory, including Feynman diagrams, as a basis for our description of the measurement process. This theory contains inverse processes that result in a non-linear dependence on the initial state which removes the von Neumann dilemma.

In the field of investigation of the measurement process, a strong belief has been established that microscopic details of the measurement interaction cannot lead to the bifurcation determining the result of measurement (see, e.g., [11,12]). This belief is based on von Neumann’s way of handling linear quantum mechanics. In this situation, many have abandoned the ambition to understand the mechanism of a single measurement and concentrated on the full ensemble of measurements. There one has studied the irreversible decoherence process that takes the initial ensemble, with μ in a pure state, into a mixed state for the final ensemble after measurement. Since 1970, the year of DeWitt’s many-worlds theory [3] and Zeh’s paper on decoherence [11], a new tradition has developed that includes different views on how to interpret quantum mechanics, see for instance Refs. [13,14]. Epistemological aspects play an important role in these interpretations.

Our idea is that the microscopic details of the measurement apparatus affect the process so that it takes μ into either of the eigenstates of the measured observable and initiates a recording of the corresponding measurement result. This is possible to see in a more developed form of linear deterministic quantum mechanics, namely the scattering theory of quantum field theory.

The development of relativistic quantum mechanics led to quantum field theory. For a situation where a quantum system μ meets a part *A* of a measurement device, interacts with it and then leaves it, scattering theory can be an adequate description. As we have pointed out already, the scattering theory of quantum field theory has the reversibility that was requested by Bell. These are our reasons for the choice of studying measurement in the scattering theory of quantum field theory.

In our approach, measurement is part of the physics studied, rather than a subject for epistemological analysis. We show how non-linearities can be generated within quantum theory. Our statistical study of measurement processes then shows that those states of the apparatus which are competitive in leading to a final state, also take μ into *one* of the eigenstates of the measured observable. Moreover, this bifurcation, leading to one of the two possible final states for μ, occurs with the frequencies given by Born’s rule.

In the following sections, we shall first give our scattering theory description and then make all possible processes subject to a statistical analysis.

## 2. The Initial Phase of Measurement as A Scattering Process

Here we study the interaction between the small system μ and a larger system *A* with a large number of degrees of freedom. The larger system is assumed to be characterized by an ensemble of possible *initial* microstates of *A*. We consider this interaction to be the first part of a measurement process.

Since we are dealing with a two-level system μ, the Pauli matrices provide a suitable formalism with $\sigma_{3}=(\begin{array}{cc} 1 & 0\\ 0 & -1 \end{array})$ representing the observable to be measured, with eigenstates ${|+\rangle }_{\mu }=(\begin{array}{c} 1\\ 0 \end{array})$ and ${|-\rangle }_{\mu }=(\begin{array}{c} 0\\ 1 \end{array})$.

Let us investigate the characteristics of the interaction between μ and *A* in scattering theory for the case with *A* in a state with (unknown) microscopic details that are summarized in a variable α. We then denote the normalized initial state of *A* by ${|0,\alpha \rangle }_{A}$ (with 0 indicating a state of preparedness). This means that we assume α to represent one microstate in an ensemble of possible initial states.

A basic requirement is that if μ is initially in the state ${|j\rangle }_{\mu }$(j=+or−), after the interaction with *A*, its state remains the same. In this process *A* changes from the initial state ${|0,\alpha \rangle }_{A}$ to a final state ${|j,\beta_{j}\left(\alpha \right)\rangle }_{A}$, also normalized. The first *j* here indicates that *A* has been marked by the state ${|j\rangle }_{\mu }$ of μ. All other characteristics of the final state of *A* are collected in $\beta_{j}\left(\alpha \right)$. The interaction thus transforms the system *A* from an initial state of readiness, characterized by α, to a final state, marked by ${|j\rangle }_{\mu }$ and characterized by $\beta_{j}\left(\alpha \right)$.

For a general normalized state of μ, ${|\psi \rangle }_{\mu }=\psi_{+}{|+\rangle }_{\mu }+\psi_{-}{|-\rangle }_{\mu }$ (with $|\psi_{+}{|}^{2}+{\left|\psi_{-}\right|}^{2}=1$), the combined initial state of μ∪A is

(1) $${|\psi \rangle }_{\mu }⊗{|0,\alpha \rangle }_{A}=\left(\psi_{+}{|+\rangle }_{\mu }+\psi_{-}{|-\rangle }_{\mu }\right)⊗{|0,\alpha \rangle }_{A}\;.$$

A measurement of $\sigma_{3}$ on μ leads to a certain result. Since two different results are possible, the μA-interaction should in general result in a transition to one of the following states,

(2) $${|+\rangle }_{\mu }⊗{|+,\beta_{+}\left(\alpha \right)\rangle }_{A}\;\;\;\;\;\;\mathrm{or}\;\;\;\;\;\;{|-\rangle }_{\mu }⊗{|-,\beta_{-}\left(\alpha \right)\rangle }_{A}\;.$$

The conclusion is then that the outcome must depend on the initial state of *A*, i.e., on α.

**Figure 1:**
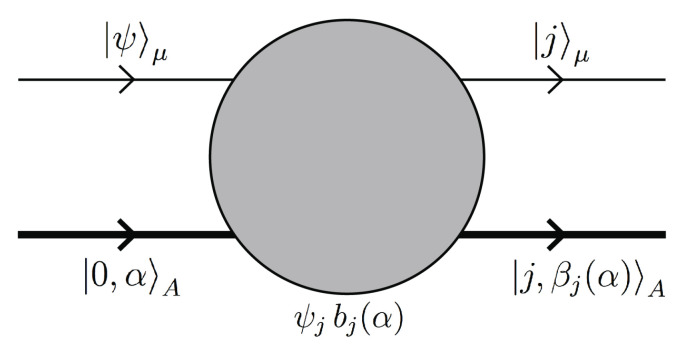
Schematic Feynman diagram for a transition from the initial state ${|\psi \rangle }_{\mu }⊗{|0,\alpha \rangle }_{A}$ to the final state ${|j\rangle }_{\mu }⊗{|j,\beta_{j}\left(\alpha \right)\rangle }_{A}$, j=±. The transition amplitude $\psi_{j}b_{j}\left(\alpha \right)$ depends on the microscopic details of the initial state ${|0,\alpha \rangle }_{A}$ of the larger system *A* and on the initial state ${|\psi \rangle }_{\mu }$ of μ.

In scattering theory, the interaction between μ and *A* is characterized by a transition operator *M*, and this leads to the (non-normalized) final state (see Figure 1),

(3) $$M{|\psi \rangle }_{\mu }⊗{|0,\alpha \rangle }_{A}=b_{+}\left(\alpha \right)\psi_{+}{|+\rangle }_{\mu }⊗{|+,\beta_{+}\left(\alpha \right)\rangle }_{A}+b_{-}\left(\alpha \right)\psi_{-}{|-\rangle }_{\mu }⊗{|-,\beta_{-}\left(\alpha \right)\rangle }_{A}\;.$$

In general, the amplitudes, $b_{+}\left(\alpha \right)$ and $b_{-}\left(\alpha \right)$, are not equal and therefore the proportions between + and − can change in a way that depends on the initial state ${|0,\alpha \rangle }_{A}$ of *A*. (Please note that *M* must not to be confused with the unitary scattering operator *S*, see Appendix A).

The requirement of a statistically unbiased measurement means that $\langle \langle |b_{+}{|}^{2}\rangle \rangle =\langle \langle |b_{-}{|}^{2}\rangle \rangle$, where 〈〈〉〉 denotes mean value over the ensemble of initial states ${|0,\alpha \rangle }_{A}$ of *A*.

Equation (3) describes a mechanism of the measurement process in which von Neumann’s dilemma is not present. Relativistic quantum mechanics, in the form of scattering theory of quantum field theory, is a more correct theory than the non-relativistic Schrödinger equation, as used in the 1930s, and we choose to use Equation (3) as our starting point.

In the Feynman-diagram language of quantum field theory, transitions between the two channels, + and −, are possible via returns to the initial state. This is a way to understand how the proportions of the channels can change as described by Equation (3). A formulation based on perturbation theory to all orders, leads to an explicitly unitary description of the whole process. (This is shown in Appendix B)

In scattering theory, transition rate (transition probability per unit time) is a central concept as we have reviewed in Appendix A. The transition rate from the initial state (1) to the final state (3) is ${\left(2\pi \right)}^{-1}w\left(\alpha \right)$, where w(α) is the squared modulus of (3),

(4) $$w\left(\alpha \right)=|\psi_{+}{|}^{2}|b_{+}{\left(\alpha \right)|}^{2}+|\psi_{-}{|}^{2}{\left|b_{-}\left(\alpha \right)\right|}^{2}\;.$$

Each term in (4) represents the partial transition rate for the corresponding channel. Because of our assumption of common mean values for $|b_{\pm }{\left(\alpha \right)|}^{2}$, the mean value of (4) is the same, $\langle \langle w\rangle \rangle =\langle \langle |b_{\pm }{|}^{2}\rangle \rangle$.

For equation (3) to properly represent a measurement process, i.e., a bifurcation that leads to a final state with μ in either of the eigenstates of $\sigma_{3}$, it is necessary that the squared moduli of the amplitudes satisfy either $|b_{+}{\left(\alpha \right)|}^{2}>>{\left|b_{-}\left(\alpha \right)\right|}^{2}$ or $|b_{-}{\left(\alpha \right)|}^{2}>>{\left|b_{+}\left(\alpha \right)\right|}^{2}$. If this holds for (almost) all microstates α in the resulting ensemble of final states, then it can function as a mechanism for the bifurcation of the measurement process.

Part of the basis for the von Neumann dilemma was the assumption that *A* is in a given initial state ${|0,\alpha \rangle }_{A}$. Before the μA-interaction, *A* can be in any of the states of the available initial ensemble. These states are ready to influence the recording process in different ways. To reach a final state, given by Equation (3), they compete with their transition rates, ${\left(2\pi \right)}^{-1}w\left(\alpha \right)$, which can differ widely between different values of α. The competition can lead to a selection and to a statistical distribution over α of the final states that is very different from the distribution in the initial ensemble.

In the following section, we will construct a mathematical model of how *A* influences the μA-interaction, including a description of the ensemble of possible initial states α. We then make a statistical analysis to show how both the bifurcation and the proper weights for the different measurement results can be understood in this simple setting.

## 3. Mathematical Model of the *μA*-interaction

We shall now model the amplitudes describing the μA-interaction, leading to the final state (3), and how it depends on the initial state ${|0,\alpha \rangle }_{A}$.

To have a generic model, we think of our system *A* as consisting of *N* independent subsystems, each interacting with μ, resulting in amplitudes that are products of *N* factors [15,16]. Since only factors resulting in differences between the two amplitudes are important, we assume

(5) $$\begin{array}{c} |b_{j}{\left(\alpha \right)|}^{2}=\prod_{n=1}^{N}\left(1+j\kappa_{n}\left(\alpha \right)\right)\;,\;\;\;\mathrm{with}\;j=+\;\mathrm{or}\;-\;, \end{array}$$

where $\kappa_{n}{\left(\alpha \right)}^{*}=\kappa_{n}\left(\alpha \right)$. Small deviations from unity in the factors are characterized by a zero mean, $\langle \langle \kappa_{n}\rangle \rangle =0$, while the independence between the subsystems is expressed by $\langle \langle \kappa_{n}\kappa_{n^{\prime }}\rangle \rangle =\delta_{nn^{\prime }}\chi^{2}$, and 0<χ<<1. We have followed the convention, used in stochastic dynamics (as for instance in Ref. [5]), to calculate to second order in $\kappa_{n}$ and then replace $\kappa_{n}\kappa_{n^{\prime }}$ by its mean $\delta_{nn^{\prime }}\chi^{2}$. This model reflects the unbiased character of the measurement device, and it guarantees that on average the squared moduli of the amplitudes are identical and equal to unity, $\langle \langle |b_{j}{\left(\alpha \right)|}^{2}\rangle \rangle =1$.

As an illustration, in Appendix C, we describe a situation where μ is a fast charged particle emerging from some process and then interacting electrically with the system *A*. Here A consists of a chain of small cylinders of ionizable material along the track where μ is passing in one of its state components. We show how the amplitude factorizes in this case.

We have chosen to keep each factor in the model close to unity in order to illustrate that even very small variations in how the subsystems interact with the system μ may result in one of the channels (one of the amplitudes), dominating over the other one, depending on the microstate α of the device. This makes it necessary to have a very large number *N* of subsystems. The critical assumptions are (i) the unbiased character of the device, i.e., not favoring any of the channels; and (ii) the independence of the subsystems of the device. The statistics of the interaction is treated in the following section.

## 4. Statistical Theory of the Transition to the Final State

We are now ready to investigate the statistical consequences of the μA-interaction modelled in the previous section. We introduce the total variance of the parameters in Equation (5), $\Xi =N\chi^{2}$, and define an aggregate variable Y=Y(α) of *A*, suitably normalized,

(6) $$\begin{array}{c} Y\left(\alpha \right)=\frac{1}{\Xi }\sum_{n=1}^{N}\kappa_{n}\left(\alpha \right)\; \end{array}$$

to represent the overall degree of enhancement/suppression (so that Y>0 for net enhancement of + and Y<0 for net enhancement of −). It follows that *Y* is characterized by mean and variance 〈〈Y〉〉=0 and $\langle \langle Y^{2}\rangle \rangle =\Xi^{-1}$. Then, for sufficiently small $\kappa_{n}$, we can rewrite (5) as

(7) $$\begin{array}{cc} |b_{j}{\left(\alpha \right)|}^{2} & =e^{\;\sum_{n}\log\left(1+j\kappa_{n}\right)}=e^{\;\Xi jY\left(\alpha \right)-\frac{1}{2}\sum_{n}\kappa_{n}^{2}}=e^{\;\Xi (j\;Y\left(\alpha \right)-\frac{1}{2})}\;, \end{array}$$

with j=+or−. Here, in the exponent, we have done the calculation to second order in $\kappa_{n}$ and we have replaced $\sum_{n}\kappa_{n}^{2}$ by $N\chi^{2}$.

Since all factors in the product (5) are independent, the distribution q(Y) over the aggregate variable Y=Y(α), defined by Equation (6), in the ensemble of initial states of *A*, is well described by the Gaussian distribution,

(8) $$\begin{array}{c} q\left(Y\right)=\sqrt{\frac{\Xi }{2\pi }}\;e^{-\frac{1}{2}\Xi Y^{2}}\;. \end{array}$$

centered around Y=0 with variance $\Xi^{-1}$.

Initial states differ in their efficiency in leading to a transition to a final state, since the total transition rate may depend strongly on α. The transition to the final state (3) with $|b_{j}{|}^{2}$ given by (7) has the rate ${\left(2\pi \right)}^{-1}w\left(Y\right)$ where

(9) $$w\left(Y\right)=|\psi_{+}{|}^{2}e^{\Xi (Y-\frac{1}{2})}+{\left|\psi_{-}\right|}^{2}e^{\Xi (-Y-\frac{1}{2})}\;,$$

with 〈〈w(Y)〉〉=1. The terms in (9) are the partial transition rates for the + and − channels.

**Figure 2:**
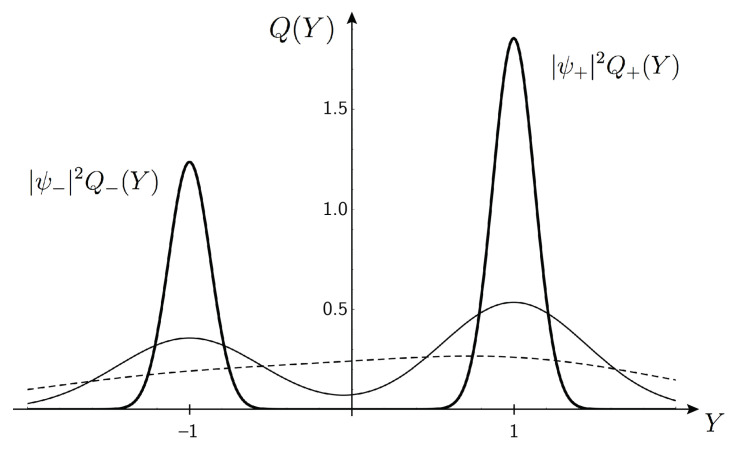
The distribution Q(Y) over *Y* of transitions taking place in μA-interaction for increasing size of *A* corresponding to Ξ=1 (broken line), Ξ=5 (thin line), and Ξ=60 (thick line). Q(Y) is composed of two distributions $Q_{+}\left(Y\right)$ and $Q_{-}\left(Y\right)$ with weights $|\psi_{+}{|}^{2}$ and $|\psi_{-}{|}^{2}$, respectively. These distributions become separated as Ξ increases. Each initial state of *A*, ${|0,\alpha \rangle }_{A}$, is represented by a certain Y=Y(α). As the size of *A* increases and Ξ becomes larger, states that are efficient in leading to a transition are found around Y=−1 and Y=+1, respectively. These initial states then lead to μ ending up in either ${|-\rangle }_{\mu }$ or ${|+\rangle }_{\mu }$, respectively, with probabilities confirming the Born rule.

The total transition rate (9) depends strongly on *Y*. We shall now go into the statistics of the final states which is strongly influenced by w(Y). To get the distribution Q(Y) over *Y* for the *final states*, corresponding to q(Y) for the initial states, we must multiply q(Y) by the transition rate (9) which is normalized in the sense that its mean value is 1. This is the standard approach in scattering theory, see, e.g., Ref. [17]. Here, it can be interpreted as a selection process, as previously discussed, that favors initial states which are efficient in leading to a transition, with a selective fitness being proportional to the transition rate (9). Thus, the distribution over final states can be written (see Figure 2)

(10) $$\begin{array}{c} Q\left(Y\right)=q\left(Y\right)w\left(Y\right)=|\psi_{+}{|}^{2}Q_{+}\left(Y\right)+{\left|\psi_{-}\right|}^{2}Q_{-}\left(Y\right)\;,\\ Q_{\pm }\left(Y\right)=\sqrt{\frac{\Xi }{2\pi }}\;e^{-\frac{1}{2}\Xi {\left(Y\mp 1\right)}^{2}}\;. \end{array}$$

The normalized partial distributions, $Q_{+}\left(Y\right)$ and $Q_{-}\left(Y\right)$, also with variance $\Xi^{-1}$, are centered around Y=1 and Y=−1 and refer to μ ending up in the state ${|+\rangle }_{\mu }$ and ${|-\rangle }_{\mu }$, respectively. The coefficients of $Q_{+}\left(Y\right)$ and $Q_{-}\left(Y\right)$ in Q(Y) are $|\psi_{+}{|}^{2}$ and $|\psi_{-}{|}^{2}$, expressing Born’s rule explicitly.

It is instructive to follow the distribution Q(Y) with growing Ξ. For small Ξ ($=N\chi^{2}$) it is broad and unimodal; it then turns broad and bimodal with narrowing peaks. For large Ξ, it is split into two well separated distributions with sharp peaks, weighted by the squared moduli of the state components of μ, $|\psi_{+}{|}^{2}Q_{+}\left(Y\right)$ and $|\psi_{-}{|}^{2}Q_{-}\left(Y\right)$, at Y=1 and Y=−1, respectively. They represent two different sub-ensembles of final states (see Equation (2)). Other values of *Y* correspond to non-competitive processes. The aggregate variable *Y* is "hidden" in the fine unknown details of *A* that can influence the μA-interaction.

From Equation (7) we see that the dominance of either channel is characterized by $|b_{+}{\left(\alpha \right)|}^{2}/{\left|b_{-}\left(\alpha \right)\right|}^{2}=e^{2\Xi Y}$ being either very large or very small. For a small system, i.e., small *N* and hence small Ξ, this ratio does not deviate much from unity which means that we have an entangled superposition of the two final states. As Ξ increases, the ratio deviates more strongly from unity and one of the channels starts to dominate. When Ξ is of the order of 10, the sub-distributions, $Q_{+}\left(Y\right)$ and $Q_{-}\left(Y\right)$, are essentially non-overlapping, Q(Y) is concentrated around ±1, and the ratio of dominance between the channels is of the order of $e^{20}\approx 10^{10}$.

The initial state for μ in (1) is a superposition, a ’*both-and* state’, and it ends up in (2) which is again a product state, with μ in *either* ${|+\rangle }_{\mu }$ *or* ${|-\rangle }_{\mu }$. The initial states of *A* vary widely in their efficiency to lead to a final state. When one transition-rate term in (9) is large, the other one is small. The selection of a large transition rate therefore also leads to a bifurcation with one of the terms in (3) totally dominating the final state.

## 5. Concluding Remarks

The main contribution in our work is that we have demonstrated that the initial stage of quantum measurement can be described within reversible quantum mechanics. The key components are (i) a scattering theory formulation with inverse processes that both guarantee unitarity and allow for a non-linear mechanism leading to the bifurcation; and (ii) a statistical analysis that reveals how initial states that are efficient in leading to a transition to a final state have a selective advantage and how this results in the correct probabilities of the measurement results as stated by Born’s rule.

In our description, we want the system *A* to be big enough for a bifurcation to take place, i.e., for $\Xi =N\chi^{2}$ to be sufficiently large. Our idea has been to follow the qualitative recipe given by Bell who formulated a principle concerning the position of the Heisenberg cut [10], i.e., the boundary of the system *A*, interacting with μ according to quantum dynamics (Ref. [10], p.124):

put sufficiently much into the quantum system that the inclusion of more would not significantly alter practical predictions

On the other hand, the system *A* should not be so large that μ∪A cannot be described by deterministic quantum mechanics. In the model that we have described, the bifurcation of measurement takes place in the reversible stage of the interaction between μ and *A* before irreversibility sets in and fixes the result. In this respect, our analysis is very different from decoherence analysis [11,18].

Beyond *A*, the measurement apparatus must be considered to be an open system with its dynamics described by a Lindblad equation [19]. The starting point for the development here is one of the final states before the Heisenberg cut, i.e., ${|+,\beta_{+}\left(\alpha \right)\rangle }_{A}$ or ${|-,\beta_{-}\left(\alpha \right)\rangle }_{A}$, in one of the sub-ensembles described by $Q_{+}\left(Y\right)$ or $Q_{-}\left(Y\right)$. Thus, the open dynamics continues only in the channel that happens to have been chosen, + or −.

For future work, a more detailed description is needed of a typical μA-interaction, including the statistics of the initial states and the selection of one state ${|0,\alpha \rangle }_{A}$ with a large transition amplitude, leading to a final state (2) with μ in one eigenstate, ${|+\rangle }_{\mu }$ or ${|-\rangle }_{\mu }$. An important task is to construct a detailed physical model of a non-biased measurement apparatus. The model of Appendix C is a beginning in this respect, but the mathematical assumptions in Equation (3) should be directly tied to physical properties of *A*. In particular, the non-bias property of *A* should be analyzed.

The system *A* should be neither too small nor too large. Then it is reasonable to describe it as mesoscopic, but Bell’s principle that we have quoted above gives no indication of its actual size. In the development of realistic models, questions of limits and the accuracy of approximations will have to be handled in more detail.

In practical scientific research, there is a common working understanding of quantum mechanics. Physicists have a common reality concept for a quantum-mechanical system when it is not observed, a kind of pragmatic quantum ontology with the *quantum-mechanical state* of the studied system as the basic concept. Development of this state in time then constitutes the quantum dynamics. If quantum mechanics now can also be used to describe the measurement process, this pragmatic quantum ontology can have a wider validity than has been commonly expected.

## Figures and Tables

**Figure A1:**
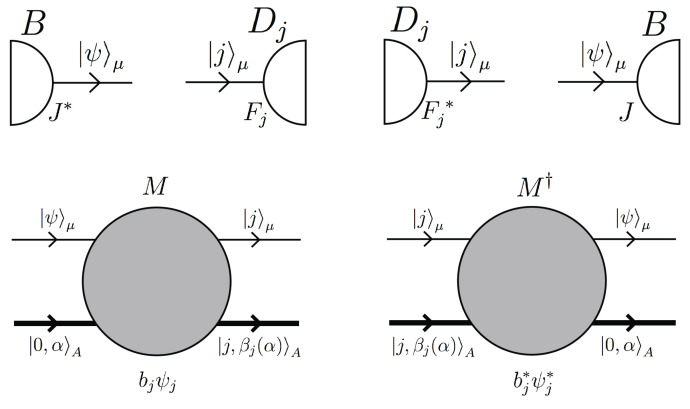
Schematic Feynman-diagram elements for the action of the source *B*, the transition matrix *M* and the sinks $D_{j}\;\left(j=\pm \right)$ and their conjugates.

**Figure A2:**
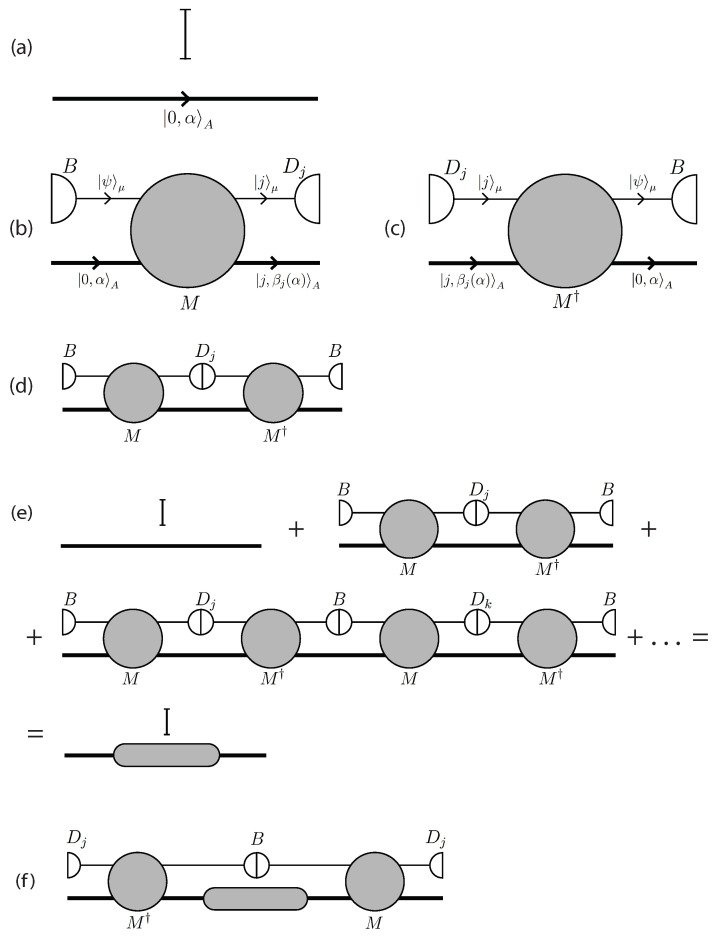
Schematic Feynman diagrams: (**a**) zero-order diagram for no change (the I-like sign above the *A*-line symbolizes "no μ-system"); (**b**) lowest order diagram for transition to a state with *A* marked by μ in the *j* state (see Figure 1); (**c**) inverse diagram of b; (**d**) diagrams b and c combined to a no-change correction; (**e**) summation over d repeated any number of times, i.e., summation of no-change diagrams to all orders; (**f**) the full perturbation expansion of the diagonal elements of the final-state density matrix with *A* marked by μ in the state ${|j\rangle }_{\mu }$.

**Figure A3:**
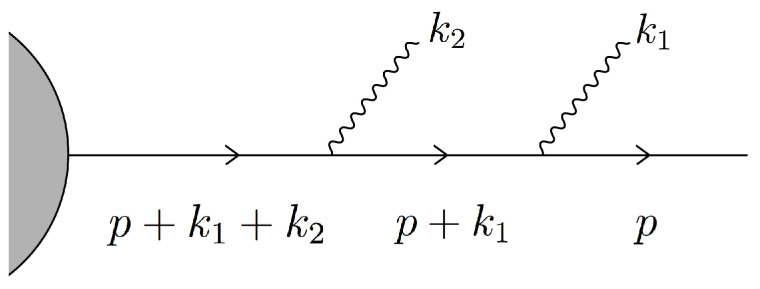
Feynman diagram for the emission of two soft photons.

## Appendix A. Scattering Matrix *S *and Transition Matrix *M*

The unitary (i.e., probability preserving) scattering operator *S*, takes an initial state |i〉 into a final state S|i〉. If a certain final state |f〉 is of interest to us then we calculate the scattering-matrix element 〈f|S|i〉. When dealing with particle scattering, it is convenient to do this in momentum space. Eigenstates of momentum are plane waves, i.e., states that occupy all space and cannot be normalized.

We shall be interested in final states |f〉 that are different from the initial state |i〉, so that |f〉 and |i〉 are orthogonal, i.e., 〈f|i〉=0, and we can replace *S* by S−1.

We use here the Quantum Electrodynamics book by Jauch and Rohrlich as our reference [17], to emphasize the development that had taken place between the physics of the 1930s and the quantum field theory of the 1950s.

To consider energy and momentum conservation, it is usual to write (Ref. [17], Equation (8)–(29))

(A1) $$\langle f|\left(S-1\right)|i\rangle =\delta \left(P_{f}-P_{i}\right)\langle f|M|i\rangle \;,$$

where $\delta (P_{f}-P_{i})$ is the 4-dimensional delta function over energy-momentum and *M* is the transition matrix.

Usually the probability for a transition into the final state |f〉, given the initial state |i〉, would be the squared modulus of (A1) but the square of a delta function does not make sense. Then one imposes a very large but finite length *L* in space and requires normalization for the wave-functions in the volume $L^{3}$, and, similarly, one imposes a time *T* for the whole process. Energy-momentum conservation is nearly exact for large *L* and *T*. One delta function in the squared modified (A1) becomes replaced by ${\left(2\pi \right)}^{-4}L^{3}T$. When normalization conventions are taken into account, the result becomes independent of *L* and proportional to *T*. After this we divide by *T* to get the transition probability per unit time (see Ref. [17], Equation (8)–(40)),

(A2) $${\left(2\pi \right)}^{-1}\delta \left(P_{f}-P_{i}\right){\left|\langle f|M|i\rangle \right|}^{2}\;.$$

Then requesting the states |i〉 and |f〉 to have the same energy and momentum, we can interpret

(A3) $${\left(2\pi \right)}^{-1}{\left|\langle f|M|i\rangle \right|}^{2}={\left(2\pi \right)}^{-1}\mathrm{Tr}\left[|f\rangle \langle f|M\rho^{\left(0\right)}M^{†}\right]\;.$$

as the transition probability per unit time, induced by *M*, from an initial state described by the density operator

(A4) $$\rho^{\left(0\right)}=|i\rangle \langle i|$$

to a final state described by the projection operator |f〉〈f|. We thus find that the transition probability-rate matrix obtained from the initial state (A4) is ${\left(2\pi \right)}^{-1}$ times

(A5) $$R=M\rho^{\left(0\right)}M^{†}\;.$$

Thus, ${\left(2\pi \right)}^{-1}R$ is the total transition rate times the density operator for the final state. Since the trace of a density operator is unity,

(A6) $${\left(2\pi \right)}^{-1}w={\left(2\pi \right)}^{-1}\;\mathrm{Tr}\;R$$

is the total transition rate. The normalized final-state density matrix is then

(A7) $$\rho^{\left(f\right)}=\frac{1}{w}R=\frac{M\rho^{\left(0\right)}M^{†}}{\mathrm{Tr}[M\rho^{\left(0\right)}M^{†}]}\;.$$

Let us consider the systems μ and *A*. *M* makes *A* entangled with μ without changing the state of μ. Still the transition amplitudes can differ between + and −. This can distort the entanglement and induce changes in the relative proportions of + and − in the final state (Equation (2)). Thus, the proportions are no longer fixed by the von Neumann dilemma; the dilemma does not arise in the scattering theory that we are considering.

## Appendix B. Calculation of Feynman Diagrams with Explicit Unitarity and Reversibility

The unitarity of the scattering matrix has not been explicitly visible in the main text. Reversibility that we have pointed out as crucial, is also not explicit. To remedy this we shall present a slightly more elaborate description of the whole process where the observed system μ is produced in its initial state ${|\psi \rangle }_{\mu }$ by an external source *B* before interaction with *A* and absorbed by a sink $D_{+}$ or $D_{-}$ in one of the possible final states after the interaction. In this version both unitarity and reversibility will be made explicit.

In this picture, the transition rate will instead be hidden and hence also the race to the final state. We therefore use the results that we have already obtained in the article, the transition rate (9) and the distribution (10) of the final states over the aggregated variable *Y*. The Born rule is also contained in (10).

As in the previous description, *A* starts in the initial state ${|0,\alpha \rangle }_{A}$ but μ is produced by *B* at an early time −T in the state ${|\psi \rangle }_{\mu }$. After μA-interaction around the time zero, μ is absorbed in an eigenstate ${|+\rangle }_{\mu }$ or ${|-\rangle }_{\mu }$ at the time +T by $D_{+}$ or $D_{-}$, leaving *A* in the state ${|+,\beta_{+}\left(\alpha \right)\rangle }_{A}$ or ${|-,\beta_{-}\left(\alpha \right)\rangle }_{A}$, respectively. We thus have one initial state ${|0,\alpha \rangle }_{A}$, a member of the ensemble of initial states, and three available final states, ${|0,\alpha \rangle }_{A}$ (no change), ${|+,\beta_{+}\left(\alpha \right)\rangle }_{A}$, and ${|-,\beta_{-}\left(\alpha \right)\rangle }_{A}$; The system μ takes part only in intermediate states.

Schematic Feynman-diagram elements for the action of the source *B*, the transition matrix *M* in Equation (3) and the sinks $D_{+}$ and $D_{-}$ are shown in Figure A1, and the factors corresponding to them, $J^{*}$, $b_{\pm }\psi_{\pm }$ and $F_{\pm }$. We represent μ by a thin line and *A* by a thick line. As in Figure 1, the interaction between μ and *A* described by the transition matrix *M*, is represented by a shaded circle. Reversibility is included through the actions of the Hermitian or complex conjugates, *J*, $M^{†}$, and $F_{j}^{*}$.

We use perturbation theory to compute the final-state density matrix,

(A8) $$\begin{array}{c} S|0,\alpha \rangle {}_{A}\;{}_{A}\langle 0,\alpha |S^{†}\;. \end{array}$$

We use the method of Nakanishi [20] to calculate this bilinear quantity directly rather than the state vector $S{|0,\alpha \rangle }_{A}$, simply because it makes normalization easy.

The diagrams of perturbation theory are shown in Figure A2. The zero-order no-change term is only an *A*-line corresponding to a contribution equal to 1 (Figure A2a). Figure A2b shows the diagram corresponding to that of Figure 1 with the source *B* and one sink $D_{j}\;\;\left(j=+,-\right)$. The inverse of this diagram is that of Figure A2c. The two taken together into one diagram represents a reduction of the no-change component due to transitions to the other states (Figure A2d). This can be repeated any number of times. All these diagrams leading back to the initial state (Figure A2e) contribute a geometrical series, representing the total no-change component of the final state,

(A9) $$\begin{array}{c} 1-\sum_{j=+,-}J\;\psi_{j}^{*}b_{j}^{*}F_{j}^{*}F_{j}b_{j}\psi_{j}J^{*}+{\left(\sum_{j=+,-}J\;\psi_{j}^{*}b_{j}^{*}F_{j}^{*}F_{j}b_{j}\psi_{j}J^{*}\right)}^{2}\pm ...=\\ \frac{1}{1+\left({|F_{+}|}^{2}{|\psi_{+}|}^{2}|b_{+}{|}^{2}+{|F_{-}|}^{2}{|\psi_{-}|}^{2}{\left|b_{-}\right|}^{2}\right){\left|J\right|}^{2}}=\frac{1}{1+{\left|J\right|}^{2}{\left|F\right|}^{2}\left({|\psi_{+}|}^{2}e^{\Xi (Y-\frac{1}{2})}+{|\psi_{-}|}^{2}e^{\Xi (-Y-\frac{1}{2})}\right)}\;. \end{array}$$

Here we have used the expressions for the amplitudes in Equation (7) and given equal strength *F* to the two sinks $D_{+}$ and $D_{-}$. The total scattering probability, i.e., the probability of *A* being marked by μ is

(A10) $$\begin{array}{c} 1-\frac{1}{1+{\left|J\right|}^{2}{\left|F\right|}^{2}\left({|\psi_{+}|}^{2}e^{\Xi (Y-\frac{1}{2})}+{|\psi_{-}|}^{2}e^{\Xi (-Y-\frac{1}{2})}\right)}=\\ J{\psi_{+}}^{*}e^{\frac{1}{2}\Xi \left(Y-\frac{1}{2}\right)}F^{*}\frac{1}{1+{\left|J\right|}^{2}{\left|F\right|}^{2}\left({|\psi_{+}|}^{2}e^{\Xi (Y-\frac{1}{2})}+{|\psi_{-}|}^{2}e^{\Xi (-Y-\frac{1}{2})}\right)}Fe^{\frac{1}{2}\Xi \left(Y-\frac{1}{2}\right)}\psi_{+}J^{*}\;+\\ J{\psi_{-}}^{*}e^{\frac{1}{2}\Xi \left(-Y-\frac{1}{2}\right)}F^{*}\frac{1}{1+{\left|J\right|}^{2}{\left|F\right|}^{2}\left({|\psi_{+}|}^{2}e^{\Xi (Y-\frac{1}{2})}+{|\psi_{-}|}^{2}e^{\Xi (-Y-\frac{1}{2})}\right)}Fe^{\frac{1}{2}\Xi \left(-Y-\frac{1}{2}\right)}\psi_{-}J^{*}\;. \end{array}$$

The two terms on the right side of (A10) are the probabilities for the final states ${|+,\beta_{+}\left(\alpha \right)\rangle }_{A}$ and ${|-,\beta_{-}\left(\alpha \right)\rangle }_{A}$, corresponding to the diagrams of Figure A1f for the remaining diagonal elements of the density matrix. For large Ξ, the no-change contribution (A9) becomes negligible. The same is true for the non-diagonal elements of the density matrix. The diagonal terms for + and − in (A10) become

(A11) $$\begin{array}{c} p_{\pm }=\frac{|\psi_{\pm }|{}^{2}e^{\pm \Xi Y}}{|\psi_{+}|{}^{2}e^{\Xi Y}+\left|\psi_{-}\right|{}^{2}e^{-\Xi Y}}\;. \end{array}$$

For Y=+1, $p_{+}=1$ and the + channel takes everything and for Y=−1, $p_{-}=1$ and the − channel takes everything. The norm is preserved, i.e., *S* is unitary. Reversibility is also clearly visible: $J^{*}$, *M* and $F_{\pm }$ are active together with their conjugates that represent inverse processes.

## Appendix C. Factorization of the Transition Amplitudes

We can think of the interaction between the small system μ and the measurement apparatus as an electromagnetic interaction with small energy and momentum transfer. In Quantum Electrodynamics (QED), it is described in terms of an exchange of soft photons. Emission and exchange of soft photons is an old and well-known example of factorizable processes in QED.

To show the factorization of soft photon exchange, we consider an outgoing electron (charge −e, mass *m*) with final momentum *p*, described by a spinor $\bar{u}\left(p\right)$ (we follow rather closely the conventions of Ref. [17]),

(A12) $$p^{2}+m^{2}=0\;,\;\;\bar{u}\left(p\right)\left(ip\cdot \gamma -m\right)=0\;,$$

after emitting two soft photons with momenta $k_{1},k_{2}$, and polarizations $\tau_{1},\tau_{2}$,

(A13) $$\begin{array}{c} k_{1}^{2}=k_{2}^{2}=0\;;\;\;\;k_{1}\cdot \tau_{1}=k_{2}\cdot \tau_{2}=0\;;\\ |k_{1}\left|\;,\;\right|k_{2}|<<m\;. \end{array}$$

In the evaluation of the Feynman diagram of Figure A3, the spinor $\bar{u}\left(p\right)$ for the outgoing electron of an original diagram (without soft photons) is replaced by an expression proportional to

(A14) $$\begin{array}{c} e^{2}\bar{u}\left(p\right)\left[\tau_{1}\cdot \gamma \frac{i(p+k_{1})\cdot \gamma +m}{{\left(p+k_{1}\right)}^{2}+m^{2}}\tau_{2}\cdot \gamma +\left(1↔2\right)\right]\frac{i(p+k_{1}+k_{2})\cdot \gamma +m}{{\left(p+k_{1}+k_{2}\right)}^{2}+m^{2}}\approx \\ e^{2}\frac{1}{2(p\cdot k_{1}+p\cdot k_{2})}\bar{u}\left(p\right)\left[\frac{\tau_{1}\cdot \gamma \left(ip\cdot \gamma +m\right)\tau_{2}\cdot \gamma \left(ip\cdot \gamma +m\right)}{2p\cdot k_{1}}+\left(1↔2\right)\right]=\\ e^{2}\frac{-p\cdot \tau_{1}p\cdot \tau_{2}}{p\cdot k_{1}+p\cdot k_{2}}\left(\frac{1}{p\cdot k_{1}}+\frac{1}{p\cdot k_{2}}\right)\bar{u}\left(p\right)=\left(s\left(k_{1}\right)\cdot \tau_{1}\right)\left(s\left(k_{2}\right)\cdot \tau_{2}\right)\bar{u}\left(p\right)\;, \end{array}$$

where we have used the basic relation for the Dirac gamma matrices, $\gamma_{\mu \nu }+\gamma_{\nu \mu }=2g_{\mu \nu }$, where $g_{\mu \nu }$ is the metric tensor. In (A14), $s_{\mu }\left(k\right)$ is the Fourier transform of the current density of a classical point charge −e moving from x=0 at time zero with the velocity $p/p_{0}$,

(A15) $$\begin{array}{c} s_{\mu }\left(k\right)=-e\frac{ip_{\mu }}{p\cdot k}=-e\int_{0}^{\infty }dt\int d^{3}x\;e^{i(k\cdot x-|k|t)}\;\delta \left(x-\frac{p}{p_{0}}t\right)\frac{p_{\mu }}{p_{0}}\;. \end{array}$$

The contribution of the diagram that μ comes from is unchanged in the limit of small $k_{1},k_{2}$. Equation (A14) states that the emission of the two photons is described by one scalar emission factor for each photon. This can be extended to also include absorption.

The case of only one photon is, of course, similar and even simpler with just one emission factor. We took the example of two photons to show how the algebra gives the two factors also in this case. For the *n*-photon case one uses the algebraic identity

(A16) $$\begin{array}{c} \sum_{\left(i_{1}i_{2}...i_{n}\right)}\frac{1}{a_{i_{1}}\left(a_{i_{1}}+a_{i_{2}}\right)...\left(a_{i_{1}}+a_{i_{2}}+...+a_{i_{n}}\right)}=\frac{1}{a_{1}a_{2}...a_{n}}\;. \end{array}$$

Thus, we can think of the classical current density

(A17) $$\begin{array}{c} j_{\mu }\left(k\right)=-e\;\delta \left(x-\frac{p}{p_{0}}t\right)\frac{p_{\mu }}{p_{0}} \end{array}$$

as representing the electron, i.e., the system μ as felt by other systems. μ can come as a very small wave-packet travelling along the $x_{1}$-axis ($p=(p_{1},0,0)$) through a substance that can be ionized. Then we take *A* to be a thin cylinder of this substance around the path of the μ wave-packet.

It is convenient to consider *A* as consisting of a chain of *N* shorter cylinders $A_{1},A_{2},...,A_{N}$ along the $x_{1}$-axis with $A_{k}$ defined by

(A18) $$\begin{array}{c} x_{1}^{\left(0\right)}+\frac{k-1}{2}\Delta l\leq x_{1}\leq x_{1}^{\left(0\right)}+\frac{k+1}{2}\Delta l\;,\\ x_{2}^{2}+x_{3}^{2}\leq \Delta r^{2}\;, \end{array}$$

where Δl and Δr are small.

For our model, we can neglect the interaction between the small cylinders and we can consider μ to interact with $A_{k}$ only during its passage, i.e., during the time interval

(A19) $$\begin{array}{c} \frac{p_{0}}{p_{1}}\left(x_{1}^{\left(0\right)}+\frac{k-1}{2}\Delta l\right)<t\leq \frac{p_{0}}{p_{1}}\left(x_{1}^{\left(0\right)}+\frac{k+1}{2}\Delta l\right)\;. \end{array}$$

Because of their independence, each of the cylinders acted on by μ via the current density (27), contributes an independent factor to the total transition rate. In the mean, the factor from the $\mu A_{k}$-interaction should not change anything but in the single case, it can contribute an unknown factor close to one, as assumed in Equation (5). Thus, we have given here a rationale for this assumption.
